# Novel Pediatric Waist-to-height Ratio Fat Mass Cutoff Predicts Liver Steatosis and Fibrosis Better than Body Mass Index: The NHANES

**DOI:** 10.1210/jendso/bvaf079

**Published:** 2025-05-03

**Authors:** Andrew O Agbaje

**Affiliations:** Institute of Public Health and Clinical Nutrition, School of Medicine, Faculty of Health Sciences, University of Eastern Finland, 70211 Kuopio, Finland; Children's Health and Exercise Research Centre, Department of Public Health and Sports Sciences, Faculty of Health and Life Sciences, University of Exeter, Exeter EX1 2LU, UK

**Keywords:** diagnosis, prevention, overweight, metabolism, obesity, pediatrics

## Abstract

**Background:**

Recent clinical consensus statements have emphasized a shift from diagnosing obesity with body mass index (BMI) requiring confirmation with surrogate markers such as waist circumference-to-height ratio (WHtR). WHtR is a highly sensitive and specific predictor of dual-energy X-ray absorptiometry-measured total body fat mass and abdominal fat mass but not lean mass. However, newly developed WHtR adiposity cut points warrant external validation before clinical application.

**Objective:**

To examine whether new WHtR cut points predict liver steatosis and fibrosis in a multiracial population.

**Methods:**

Data from 6464 (54% female) multiracial US participants from the National Health and Nutrition Examination Survey conducted between 2021 and 2023 were analyzed. Liver fibrosis was assessed with transient elastography and staged as fibrosis stage F0 to F4 and liver steatosis graded S0 to S3.

**Results:**

Participants' mean (SD) age was 47.3 (20.9) years. The prevalence of WHtR cut points of 0.40 to <0.50 (normal fat mass), 0.5 to <0.53 (high fat mass), and ≥0.53 (excess fat mass) was 20.3%, 13.6%, and 64.5%. Multivariable-adjusted WHtR high fat mass predicted liver steatosis (odds ratio 1.63 [95% confidence interval 1.16-2.29] *P* = .005) and fibrosis (1.31 [1.01-1.70] *P* = .043). Excess WHtR fat mass was associated with liver steatosis (4.02 [2.87-5.64] *P* < .001) and fibrosis (1.61 [1.03-2.54] *P* = .038). Normal WHtR fat mass predicted lower odds of liver steatosis (0.52 [0.37-0.73] *P* < .001) and fibrosis/cirrhosis (0.48 [0.30-0.76] *P* < .001). WHtR high fat mass and excess fat mass separately predicted higher odds of liver steatosis 1.6-fold and 6-fold, respectively, better than BMI-overweight and BMI-obesity.

**Conclusion:**

The simple and universally accessible WHtR cut points may be useful in clinical and public health practice for obesity screening, diagnosis, and management.

Obesity is a relapsing metabolic disease driven by excess adiposity or fat accumulation [1-8]. Recent consensus expert statements and clinical guidelines, such as the Lancet Commission on redefining and diagnosing obesity and the European Association for the Study of Obesity's framework for diagnosis, staging, and management of obesity, have highlighted the well-known limitations of body mass index (BMI) in accurately assessing excess adiposity [1, 2, 9]. These BMI limitations include the inability to distinguish between fat mass and muscle mass; not accounting for regional fat distribution; variability in age, sex, and ethnicity; under- or overdiagnosis of obesity; U-shaped mortality prediction, etc. [1, 2].

The Lancet Commission statement concluded that the clinical assessment of obesity requires confirmation of excess/abnormal adiposity by a direct body fat measurement, such as dual-energy X-ray absorptiometry (DEXA), or by at least 1 anthropometric criteria such as waist circumference-to-height ratio (WHtR) in addition to BMI [1]. Apart from a waist-to-height ratio threshold of >0.5, other cut points that correspond to the severity of excess fat that could be useful in clinical obesity diagnosis were missing in the consensus statements due to limited evidence [1, 2, 10-12]. A limitation of the current waist-to-height ratio cut points is that it is arbitrarily derived based on ease of memorization [13, 14]. For example, the waist-to-height ratio cut point in the UK's National Institute for Health and Care Excellence guideline for children and adolescents includes waist-to-height ratio values that are rare but common in adults [13]. The National Institute for Health and Care Excellence guideline states that “healthy central adiposity: waist-to-height ratio 0.4 to 0.49, indicates no increased health risk, an increased central adiposity: waist-to-height ratio 0.5 to 0.59, indicates increased health risk, and high central adiposity: waist-to-height ratio 0.6 or more, indicating further increased health risk” [13]. Until recently, there have been no objectively derived pediatric-specific cutoffs predicting both total body and/or central adiposity [15].

A recent study in >7000 healthy British children followed up until young adulthood revealed waist-to-height ratio cut points that longitudinally correspond to DEXA-measured excess fat at the 85th and 95th percentiles [16]. These waist-to-height ratio cut points identified 0.50 for males and 0.51 for females at the 85th percentile (high fat mass) and 0.53 for males and 0.54 for females at the 95th percentile (excess fat mass) [16]. Although a freely accessible waist-to-height ratio calculator based on the cut point has been developed (https://urfit-child.com/waist-height-calculator/), the predictive ability of the cut points remains untested in a large multiracial population, hence limiting its clinical application globally [1-3, 10, 11, 16, 17]. BMI is known to differ by race, and evidence suggests that waist-to-height ratio is universally consistent, but further confirmation is warranted [1, 14]. Recently, the waist-to-height ratio cut points were externally validated as predictors of prediabetes and type 2 diabetes [15]. The current study therefore validates the predictive ability of the newly developed waist-to-height ratio cut points to detect the risk of liver steatosis and liver fibrosis/cirrhosis in a multiracial US population drawn from the latest National Health and Nutrition Examination (NHANES) survey conducted between 2021 and 2023.

## Methods

This study included participants from the NHANES, which is a nationally representative study conducted by the National Center for Health Statistics of the Centers for Disease Control and Prevention to assess the health and nutritional status of adults and children in the United States. The study design and methods have been described in detail previously (https://www.cdc.gov/nchs/nhanes/about/index.html). The survey protocol was approved by the Research Ethics Review Board of the National Center for Health Statistics. Written informed consent from all participants was obtained by NHANES. This cohort study included children and adolescents 12 years of age or older who had liver scan assessments during the most recent NHANES cycle conducted during the COVID pandemic from August 2021 to August 2023. Altogether, 11 933 participated in the NHANES survey, while 6794 participants had liver scans but only 6464 participants who had complete assessments of waist-to-height ratio and liver scan assessments were included. The original data generated and analyzed during this study are included in the Center for Diseases Control and Prevention data repository [18].

### Anthropometric Measures

NHANES collected height, weight, and waist circumference via the Mobile Examination Center by trained health technicians. The participant's age at the time of the screening interview determined the body measures examination protocol. BMI was calculated as weight in kilograms divided by height in meters squared. BMI-assessed normal weight, overweight, and obesity in >19-year-old participants were categorized as <25 kg/m^2^, ≥ 25 kg/m^2^, and ≥30 kg/m^2^, respectively. For participants aged <19 years, the Centers for Disease Control growth chart of >85th to <95th percentile was categorized as overweight and ≥95th percentile as obesity. Waist-to-height ratio was calculated as waist circumference in centimeters divided by height in centimeters. The waist-to-height ratio is a highly sensitive and specific predictor of DEXA-measured total body fat mass and trunk (abdominal) fat mass [15, 16]. Waist-to-height ratio cut points are <0.40 (low fat mass), 0.40 to < 0.50 (normal fat mass), 0.50 to < 0.53 (high fat mass), and ≥0.53 (excess fat mass) in males [16]. Waist-to-height ratio cut points for females are <0.40 (low fat mass), 0.40 to < 0.51 (normal fat mass), 0.51 to < 0.54 (high fat mass), and ≥0.54 (excess fat mass) [15, 16].

### Cardiometabolic Assessment

Systolic and diastolic blood pressure and heart rate were measured 3 times consecutively and 60 seconds apart after participants had been seated for 5 minutes using a digital upper-arm electronic blood pressure measurement device, Omron HEM–907XL. Serum blood samples for total cholesterol and high-sensitivity C-reactive protein were collected and analyzed with a Roche/Hitachi Cobas 8000 analyzer series, a fully automated, high-throughput laboratory system for clinical chemistry and immunology tests. A Cobas c311 analyzer was used for serum glucose analysis. A 2-site immunoenzymometric assay was performed on serum insulin using the Tosoh AIA System analyzers NHANES (RRID:SCR_013201).

### Demographic and Lifestyle Assessments

The family and sample person demographics questionnaires were administered by trained interviewers using the Computer-Assisted Personal Interview system in the participant's home or by telephone using either English or Spanish. Participants 16 years and older and emancipated minors were interviewed directly. Data on sex, age, educational status, income and savings history, race, alcohol use, smoking status, and previous diagnosis of coronary heart disease and type 2 diabetes were collected via questionnaires. Sedentary time and physical activity were collected using a physical activity questionnaire. Questions regarding sitting at school and at home, getting to and from places, or with friends, including time spent sitting at a desk, traveling in a car or bus, reading, playing cards, watching television, or using a computer on a typical day were asked to ascertain time spent sedentary. Questions on how much time is spent on moderate-intensity activities that can cause moderate increases in breathing or heart rate were asked.

### Liver Assessment

Liver fibrosis was measured by FibroScan®, which uses ultrasound and vibration-controlled transient elastography to derive liver stiffness and the controlled attenuation parameter assessed liver steatosis [19-21]. The elastography measurements were obtained in the NHANES Mobile Examination Center using the FibroScan model 502 V2 Touch equipped with a medium or extra-large wand (probe). The elastography exam was performed by NHANES examiners, including health technicians, radiology technicians, and clinicians (registered nurses), who were trained and certified by NHANES staff and the equipment manufacturer (Echosens^TM^ North America). The exams were performed according to the manufacturer's guidelines. A complete examination implies that participants had fasted for at least 3 hours, had 10 or more complete stiffness measures, and a liver stiffness interquartile range / median complete stiffness < 30%. Cut-off values for controlled attenuation parameter (CAP) score for different grades of steatosis (S0-S3) were derived from a meta-analysis on CAP technology: S0 was defined as a score of less than 248 dB/m (<10% steatosis); S1 as a score of 248 to less than 268 dB/m [10– < 33% steatosis (mild)]; S2 as a score of 268 to less than 280 dB/m [33– < 66% steatosis (moderate)]; and S3 as a score of 280 dB/m or more [≥66% steatosis (severe)] [21]. CAP scores of 248 dB/m or greater (≥S1) were considered as suspected steatosis. Participants' CAP scores were considered eligible for analysis if 10 valid readings (100-400 dB/m) could be obtained [19].

### Statistical Analysis

Participants’ descriptive characteristics were summarized as means and SD or frequencies and percentages. Differences in waist-to-height ratio categories across race and age groups were examined with a univariate general linear model. The associations presented as the odds ratio of the different waist-to-height ratio cut points and the risk of liver fibrosis and steatosis were examined with generalized linear mixed-effect models with a logit link for the total cohort. The optimal model with the lowest Bayesian information criteria was that with sex as a main effect, a random intercept modeled for the participants, and subject cluster to account for inter-individual correlations. The specified random effect covariance type is the variance component. While the generalized linear mixed-effect model with a full information maximum likelihood is robust for handling missing at random covariate data, we elected to additionally conduct 20 cycles of multiple imputations to account for missing covariates. Little's missing completely at random test was conducted to ascertain data missingness and concluded that covariates were not missing completely at random. Regression-modeled multiple imputations were conducted with 20 cycles of imputation with 10 iterations generating 20 imputed data sets and the specified constraints for the imputation process were the observed minimum and maximum values. Model 1 was unadjusted. Model 2 was adjusted for age, sex, systolic blood pressure, heart rate, educational status, smoking status, race, sedentary time, moderate physical activity, and fasting insulin, glucose, total cholesterol, and high-sensitivity C-reactive protein. Race-based and age-group-based statistical analyses were also reported separately. The analyses were repeated with BMI categories as predictors of liver steatosis and fibrosis according to age group due to differences in BMI categories per age (≤19 years and >19 years old). Model 1 and model 2 are as described earlier with an additional model 3 mutually adjusted for waist-to-height ratio. For the participants >19 years old, a similar waist-to-height ratio category in relation to liver steatosis analyses with mutual adjustment for BMI was conducted to compare the predictive ability of BMI and waist-to-height ratio. Multiple testings were corrected with Sidak's correction. All statistical analyses were performed using SPSS statistics software, Version 29.0 (IBM Corp., Armonk, NY, USA).

## Results

### Cohort Study Characteristics

**Figure 1.**
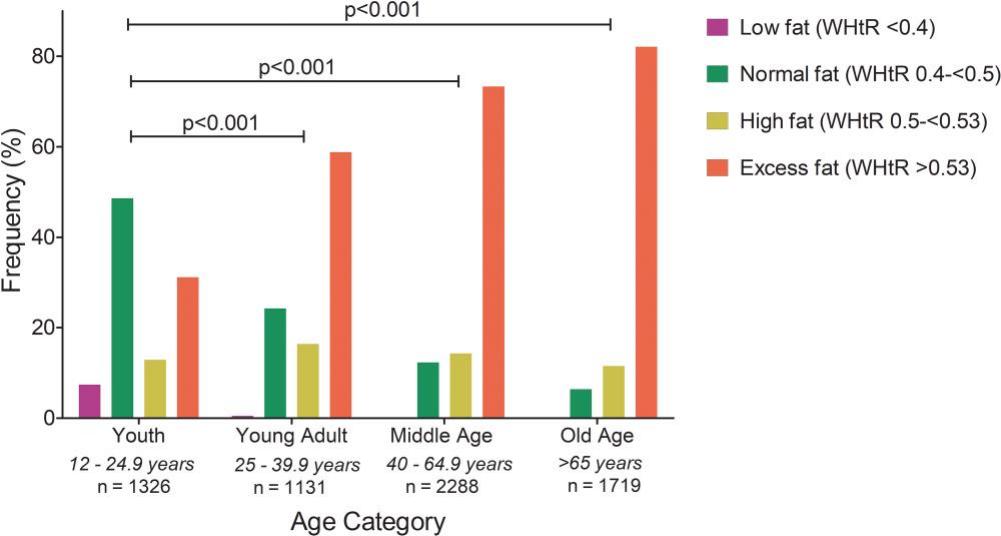
Waist circumference-to-height ratio fat mass categories by age group. Data was drawn from the US National Health and Nutrition Examination Survey (conducted between August 2021 and August 2023). Altogether, 6464 participants were included in the analysis. *P*-values <.05 were considered statistically significant. A univariate general linear model was applied to analyze between-group differences. The mean (SD) age of the included participants was 47.3 (20.9) years.

**Figure 2.**
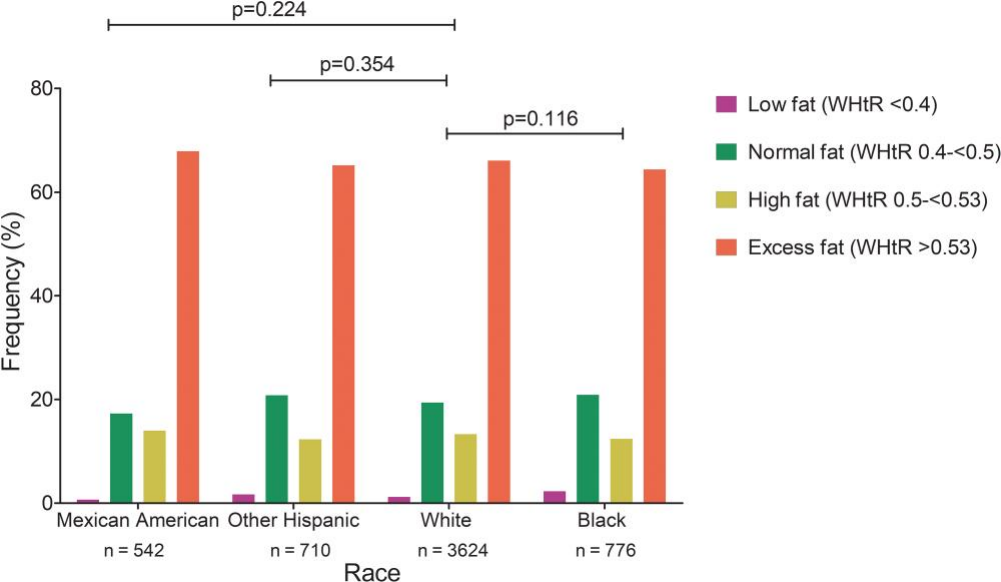
Waist circumference-to-height ratio fat mass categories by race. Data was drawn from the US National Health and Nutrition Examination Survey (conducted between August 2021 and August 2023). Altogether, 6464 participants were included in the analysis. *P*-values <.05 were considered statistically significant. A univariate general linear model was applied to analyze between-group differences. The mean (SD) age of the included participants was 47.3 (20.9) years.

Among 6464 US participants, 12% were non-Hispanic Black, 8.4% were Mexican American, and 56% were non-Hispanic White. The mean (SD) age was 47.3 (20.9) years, while 20.5% were between ages 12 to 24.9 years and 26.6% were >65 years old (Table 1). In the whole cohort, the prevalence of a waist-to-height ratio cut point of 0.40 to <0.50 (normal fat mass), 0.5 to 0.53 (high fat mass), and >0.53 (excess fat mass) was 20.3%, 13.6%, and 64.5%, respectively, while the prevalence of liver steatosis (S1-S3) was 12% and liver fibrosis/cirrhosis (F2-F4) was 27% (Table 1). Waist-to-height ratio cut-off frequency significantly differs by age group with >65-year-olds having more excess fat mass compared to 12- to 24.9-year-olds (Fig. 1). There were no statistically significant differences between waist-to-height ratio cut points frequency according to race (Fig. 2). Other characteristics are described in Table 1.

**Table 1.** Descriptive characteristics of NHANES cohort participants during the survey of 2021-2023

Variables	n = 6464 Mean (SD)
Demography	
Age at screening (years), n (%)	47.25 (20.94)
12-24.9	1326 (20.5)
25-39.9	1131 (17.5)
40-64.9	2289 (35.4)
≥65	1718 (26.6)
Sex (female), n (%)	3471 (53.7)
Race, n (%) Non-Hispanic White	3625 (56.1)
Non-Hispanic Black	776 (12.0)
Mexican American	542 (8.4)
Other Hispanic	710 (11.0)
Other race, including multiracial	811 (12.5)
Anthropometry and body composition	
Height, cm	167 (10.1)
Weight, kg	80.28 (22.1)
Waist circumference, cm	97.78 (17.76)
Waist circumference-to-height ratio	0.59 (0.11)
Waist-to-height ratio category <0.40 (low fat mass)	106 (1.6)
0.40-< 0.50 (normal fat mass)	1309 (20.3)
0.50-< 0.53 (high fat mass)	882 (13.6)
≥0.53 (excess fat mass)	4166 (64.5)
Body mass index (kg/m^2^)	28.76 (7.19)
Overweight in ≤ 19-year-olds, n (%)	179 (17.2)
Obesity in ≤ 19-year-olds, n (%)	247 (23.8)
Overweight in >19-year-olds, ≥25 kg/m^2^, n (%)	1788 (32.4)
Obesity in >19-year-olds, ≥30 kg/m^2^, n (%)	2198 (39.8)
Fasting plasma metabolic indices	
High-density lipoprotein, mmol/L	1.45 (0.35)
Total cholesterol	4.58 (0.93)
Glucose, mmol/L	5.97 (1.76)
Insulin, mU/L	14.24 (24.14)
High-sensitivity C-reactive protein, mg/L	3.81 (7.15)
Vascular measures	
Heart rate, beats/min	72 (12)
Systolic blood pressure, mmHg	121 (18)
Diastolic blood pressure, mmHg	73 (11)
Liver indices	
Liver stiffness assessment, kPA	4.90 (2.20)
Significant or advanced liver fibrosis, ≥7.9-< 11.7 kPa, n (%)	458 (7.1)
Liver cirrhosis, ≥11.7 kPa, n (%)	315 (4.9)
Liver fat (CAP value), dB/m	255.74 (63.91)
Suspected liver steatosis, ≥248-< 280 dB/m, n (%)	1121 (26.1)
Severe liver steatosis, ≥280 dB/m, n (%)	39 (0.9)
Lifestyle factors	
Alcohol use (ever have 4/5 or more drinks every day)	468 (18.4)
Smoking status (Do you now smoke cigarettes?)	389 (32.4)
Diagnosed with coronary heart disease	101 (3.5)
Diagnosed with type 2 diabetes mellitus	326 (8.6)
Educational status (high school and college graduates)	4763 (73.7)
Income (savings of >$20 000), n (%)	1504 (42.9)
Sedentary (min/day)	413 (731)
Moderate physical activity (min/day)	79 (396)

The values are means (SDs) and median (interquartile range), except for lifestyle factors and race in frequencies and percentages. In <19-year-olds, overweight (85th to <95th percentile) and obesity (≥95th percentile) cut-off criteria are based on the Centers for Disease Control growth chart.

Abbreviations: NHANES, National Health and Nutrition Examination Survey.

### Association of Waist-to-height Ratio Cut Points With the Risk of Liver Diseases

Relative to low fat mass waist-to-height ratio category, the normal fat mass waist-to-height ratio category predicted lower odds of liver steatosis (0.52 [0.37-0.73] *P* < .001) and fibrosis/cirrhosis (0.48 [0.30-0.76] *P* < .001) in a fully adjusted model (Table 2). High fat mass waist-to-height ratio category was associated with higher odds of liver steatosis (odds ratio 1.63 [95% confidence interval 1.16-2.29] *P* = .005) and fibrosis (1.31 [1.01-1.70] *P* = .043). Excess fat mass waist-to-height ratio category was associated with liver steatosis (4.02 [2.87-5.64] *P* < .001) and fibrosis (1.61 [1.03-2.54] *P* = .038) (Table 2). These results were consistent despite applying the female waist-to-height ratio cut points to the whole cohort (Table 3).

**Table 2.** Associations of WHtR male cut points with liver steatosis and cirrhosis in the total cohort

n = 6464	Liver steatosis (S1-S3)		Liver fibrosis (F2-F4)	
	Odds ratio (95% CI)	*P*-value	Odds ratio (95% CI)	*P*-value
WHtR (<0.40), low fat mass				
	Reference		Reference	
WHtR (0.40-< 0.50), normal fat mass				
Model 1	0.39 (0.35-0.43)	**<.001**	1.51 (1.17-1.95)	**.002**
Model 2	0.52 (0.37-0.73)	**<.001**	0.48 (0.30-0.76)	**.002**
WHtR (0.50-< 0.53), high fat mass				
Model 1	0.77 (0.69-0.86)	**<.001**	1.85 (1.43-2.39)	**<.001**
Model 2	1.63 (1.16-2.29)	**.005**	1.31 (1.01-1.70)	**.043**
WHtR (≥0.53), excess fat mass				
Model 1	2.21 (1.99-2.45)	**<.001**	6.58 (5.12-8.46)	**<.001**
Model 2	4.02 (2.87-5.64)	**<.001**	1.61 (1.03-2.54)	**.038**

Model 1 was unadjusted. Model 2 was adjusted for age, sex, systolic blood pressure, heart rate, educational status, smoking status, race, sedentary time, moderate physical activity, and fasting insulin, glucose, total cholesterol, and high-sensitivity C-reactive protein. Odds ratios were computed from the generalized linear mixed-effect model with logit-link. A 2-sided *P*-value < .05 was considered statistically significant and are bolded. Multiple testing was corrected with Sidak's correction. Multiple imputations were used to account for missing variables. Categorical predictor level relative to the reference category is associated with the odds of the outcome. Transient elastography cut-off values were used for fibrosis staging [F0-F4: F0-F1, < 7.9 kPa; F2, 7.9-<8.8 kPa; F3, 8.8-<11.7 kPa; and F4 (cirrhosis), ≥ 11.7 kPa]. Cut-off values for controlled attenuation parameter score for different grades of steatosis (S0-S3) were derived from a meta-analysis on controlled attenuation parameter technology: S0 was defined as a score of less than 248 dB/m (<10% steatosis); S1 as a score of 248 to less than 268 dB/m [10– < 33% steatosis (mild)]; S2 as a score of 268 to less than 280 dB/m [33– < 66% steatosis (moderate)]; and S3 as a score of 280 dB/m or more [ ≥ 66% steatosis (severe)].

Abbreviations: CI, confidence interval; WHtR, waist circumference-to-height ratio.

**Table 3.** Associations of WHtR female cut points with liver steatosis and cirrhosis in the total cohort

n = 6464	Liver steatosis (S1-S3)		Liver fibrosis (F2-F4)	
	Odds ratio (95% CI)	*P*-value	Odds ratio (95% CI)	*P*-value
WHtR (<0.40), low fat mass				
	Reference		Reference	
WHtR (0.40-< 0.51), normal fat mass				
Model 1	0.41 (0.37-0.46)	**<.001**	1.58 (1.22-2.04)	**<.001**
Model 2	0.60 (0.43-0.84)	**.003**	0.50 (0.32-0.79)	**.003**
WHtR (0.51-< 0.54), high fat mass				
Model 1	0.88 (0.79-0.99)	**.027**	1.79 (1.38-2.32)	**<.001**
Model 2	1.85 (1.31-2.59)	**<.001**	1.12 (0.85-1.47)	.170
WHtR (≥0.54), excess fat mass				
Model 1	2.24 (2.02-2.49)	**<.001**	6.58 (5.12-8.46)	**<.001**
Model 2	4.03 (2.87-5.65)	**<.001**	1.61 (1.03-2.53)	**.04**

Model 1 was unadjusted. Model 2 was adjusted for age, sex, systolic blood pressure, heart rate, educational status, smoking status, race, sedentary time, moderate physical activity, and fasting insulin, glucose, total cholesterol, and high-sensitivity C-reactive protein. Odds ratios were computed from the generalized linear mixed-effect model with logit-link. A 2-sided *P*-value < .05 was considered statistically significant and are bolded. Multiple testing was corrected with Sidak's correction. Multiple imputations were used to account for missing variables. Categorical predictor level relative to the reference category is associated with the odds of the outcome. Transient elastography cut-off values were used for fibrosis staging [F0-F4: F0-F1, < 7.9 kPa; F2, 7.9-<8.8 kPa; F3, 8.8-<11.7 kPa; and F4 (cirrhosis), ≥ 11.7 kPa]. Cut-off values for controlled attenuation parameter score for different grades of steatosis (S0-S3) were derived from a meta-analysis on controlled attenuation parameter technology: S0 was defined as a score of less than 248 dB/m (<10% steatosis); S1 as a score of 248 to less than 268 dB/m [10– < 33% steatosis (mild)]; S2 as a score of 268 to less than 280 dB/m [33– < 66% steatosis (moderate)]; and S3 as a score of 280 dB/m or more [ ≥ 66% steatosis (severe)].

Abbreviations: CI, confidence interval; WHtR, waist circumference-to-height ratio.

Among 12- to 24.9-year-old participants, high and excess fat mass waist-to-height ratio categories were significantly associated with the odds of higher liver steatosis but not liver fibrosis (Table 4). However, among ≥65-year-old participants, the excess fat mass waist-to-height ratio category was associated with higher odds of liver steatosis and fibrosis (Table 4). Among the minority racial groups (non-Hispanic Blacks, Mexican Americans, and other Hispanics), the excess fat mass waist-to-height ratio category was associated with higher odds of liver steatosis and fibrosis (Table 5).

**Table 4.** Youth and elderly age group stratified association of WHtR categories with liver steatosis and cirrhosis

	Liver steatosis (S1-S3)		Liver fibrosis (F2-F4)	
	Odds ratio (95% CI)	*P*-value	Odds ratio (95% CI)	*P*-value
Age 12-24.9 years youth category (n = 1326)				
Low fat mass (WHtR <0.40) n = 98	Reference		Reference	
Normal fat mass (WHtR 0.40-< 0.50), n = 644	1.28 (0.36 -4.48)	.699	1.29 (0.30-5.53)	.729
High fat mass (WHtR 0.50-< 0.53), n = 171	8.97 (2.06-39.04)	**.003**	1.96 (0.37-10.28)	.429
Excess fat mass (WHtR ≥0.53), n = 413	138.3 (34.4-556.2)	**<.001**	2.82 (0.66-12.03)	.163
Age >65 years elderly category (n = 1718)				
Normal fat mass (WHtR 0.40-< 0.50), n = 110	Reference		Reference	
High fat mass (WHtR 0.50-< 0.53), n = 197	4.19 (1.10-15.92)	**.035**	0.64 (0.17-2.40)	.507
Excess fat mass (WHtR ≥0.53), n = 1411	25.54 (8.20-79.53)	**<.001**	3.84 (1.30-11.36)	**.015**

Model was adjusted for sex, systolic blood pressure, heart rate, educational status, smoking status, race, sedentary time, moderate physical activity, and fasting insulin, glucose, total cholesterol, and high-sensitivity C-reactive protein. There were no recorded fasting blood samples for >65-year-olds, and thus blood samples were not controlled for in their analyses. There were no participants at >65 years with WHtR less than 0.4. Odds ratios were computed from the generalized linear mixed-effect model with logit-link. A 2-sided *P*-value < .05 was considered statistically significant and are bolded. Multiple testing was corrected with Sidak's correction. Multiple imputations were used to account for missing variables. Categorical predictor level relative to the reference category is associated with the odds of the outcome. Transient elastography cut-off values were used for fibrosis staging [F0-F4: F0-F1, < 7.9 kPa; F2, 7.9-<8.8 kPa; F3, 8.8-<11.7 kPa; and F4 (cirrhosis), ≥ 11.7 kPa]. Cut-off values for controlled attenuation parameter score for different grades of steatosis (S0-S3) were derived from a meta-analysis on controlled attenuation parameter technology: S0 was defined as a score of less than 248 dB/m (<10% steatosis); S1 as a score of 248 to less than 268 dB/m [10- < 33% steatosis (mild)]; S2 as a score of 268 to less than 280 dB/m [33-< 66% steatosis (moderate)]; and S3 as a score of 280 dB/m or more [ ≥ 66% steatosis (severe)].

Abbreviations: CI, confidence interval; WHtR, waist circumference-to-height ratio.

**Table 5.** Race-based association of WHtR categories with liver steatosis and cirrhosis

	Liver steatosis (S1-S3)		Liver fibrosis (F2-F4)	
	Odds ratio (95% CI)	*P*-value	Odds ratio (95% CI)	*P*-value
Non-Hispanic Black (n = 776)				
Normal fat mass (WHtR 0.40-< 0.50)	Reference		Reference	
High fat mass (WHtR 0.50-< 0.53)	3.38 (0.46-24.74)	.231	0.29 (0.03-3.32)	.323
Excess fat mass (WHtR ≥0.53)	32.82 (6.39-168.6)	**<.001**	4.87 (1.09-21.76)	**.038**
Mexican American (n = 542)				
Normal fat mass (WHtR 0.40-< 0.50)	Reference		Reference	
High fat mass (WHtR 0.50-< 0.53)	6.32 (1.03-38.59)	**.046**	0.44 (0.06-3.03)	.401
Excess fat mass (WHtR ≥0.53)	417.8 (88.3-1976.6)	**<.001**	3.91 (1.03-14.82)	**.045**
Other Hispanic (n = 710)				
Normal fat mass (WHtR 0.40-< 0.50)	Reference		Reference	
High fat mass (WHtR 0.50-< 0.53)	7.27 (1.49-35.55)	**.014**	0.49 (0.37-6.68)	.595
Excess fat mass (WHtR ≥0.53)	314.2 (75.86-1301.7)	**<.001**	5.49 (1.05-28.79)	**.044**

Model was adjusted for age, sex, systolic blood pressure, heart rate, educational status, smoking status, sedentary time, moderate physical activity, and fasting insulin, glucose, total cholesterol, and high-sensitivity C-reactive protein. Odds ratios were computed from the generalized linear mixed-effect model with logit-link. A 2-sided *P*-value < .05 was considered statistically significant and are bolded. Multiple testing was corrected with Sidak's correction. Multiple imputations were used to account for missing variables. Categorical predictor level relative to the reference category is associated with the odds of the outcome. Transient elastography cut-off values were used for fibrosis staging [F0-F4: F0-F1, < 7.9 kPa; F2, 7.9-<8.8 kPa; F3, 8.8-<11.7 kPa; and F4 (cirrhosis), ≥ 11.7 kPa]. Cut-off values for controlled attenuation parameter score for different grades of steatosis (S0-S3) were derived from a meta-analysis on controlled attenuation parameter technology: S0 was defined as a score of less than 248 dB/m (<10% steatosis); S1 as a score of 248 to less than 268 dB/m [10– < 33% steatosis (mild)]; S2 as a score of 268 to less than 280 dB/m [33- < 66% steatosis (moderate)]; and S3 as a score of 280 dB/m or more [ ≥ 66% steatosis (severe)].

Abbreviations: CI, confidence interval; WHtR, waist circumference-to-height ratio.

### Association of BMI Cut Points With the Risk of Liver Diseases

Among ≤19-year-olds, relative to the normal weight category, the overweight category predicted higher odds of liver steatosis (2.47 [2.26-2.70] *P* < .001) but not fibrosis/cirrhosis (1.02 [.93-1.11] *P* = .726) in a fully adjusted model (Table 6). Further adjustment for waist-to-height ratio significantly diminished the strength of the association between overweight and liver steatosis by 1.6-fold (1.37 [1.23-1.53] *P* < .001). Relative to the normal weight category, the obesity category predicted higher odds of liver steatosis (4.61 [4.25-4.99] *P* < .001) and fibrosis/cirrhosis (1.16 [1.07-1.26] *P* < .001) in a fully adjusted model in ≤ 19-year-olds (Table 6). Further adjustment for waist-to-height ratio significantly attenuated the strength of the association between obesity and liver steatosis by 3.3-fold (1.40 [1.20-1.62] *P* < .001), while the association between obesity and liver fibrosis was reversed (.85 [0.73-0.99] *P* = .038), suggestive of a protective relationship (Table 6).

**Table 6.** Associations of body mass index category cut points with liver steatosis and cirrhosis in youth and adults

		Liver steatosis (S1-S3)		Liver fibrosis (F2-F4)	
		Odds ratio (95% CI)	*P*-value	Odds ratio (95% CI)	*P*-value
Youth ≤19 years old (n = 1038)					
Model 1	Normal weight (n = 612)	Reference		Reference	
	Overweight (n = 179)	2.42 (2.22-2.63)	**<.001**	1.00 (0.92-1.09)	.924
	Obesity (n = 247)	4.77 (4.43-5.15)	**<.001**	1.22 (1.13-1.31)	**<.001**
Model 2	Normal weight	Reference		Reference	
	Overweight	2.47 (2.26-2.70)	**<.001**	1.02 (0.93-1.11)	.726
	Obesity	4.61 (4.25-4.99)	**<.001**	1.16 (1.07-1.26)	**<.001**
Model 3	Normal weight	Reference		Reference	
	Overweight	1.37 (1.23-1.53)	**<.001**	0.87 (0.78-0.97)	**.013**
	Obesity	1.40 (1.20-1.62)	**<.001**	0.85 (0.73-0.99)	**.038**
Adults >19 years old (n = 5426)					
Model 1	Normal weight (n = 1477)	Reference		Reference	
	Overweight (n = 1778)	9.07 (6.80-12.10)	**<.001**	1.88 (1.26-2.80)	.002
	Obesity (n = 2171)	24.51 (18.99-31.63)	**<.001**	17.38 (11.93-25.33)	**<.001**
Model 2	Normal weight	Reference		Reference	
	Overweight	8.23 (6.18-10.96)	**<.001**	1.63 (1.07-2.47)	.022
	Obesity	21.98 (16.95-28.51)	**<.001**	15.01 (10.09-22.31)	**<.001**
Model 3	Normal weight	Reference		Reference	
	Overweight	4.13 (2.99-5.72)	**<.001**	0.31 (0.19-0.71)	**<.001**
	Obesity	4.77 (3.14-7.26)	**<.001**	0.37 (0.19-0.71)	**.003**

Model 1 was unadjusted. Model 2 was adjusted for age, sex, systolic blood pressure, heart rate, educational status, smoking status, race, sedentary time, moderate physical activity, and fasting insulin, glucose, total cholesterol, and high-sensitivity C-reactive protein. Model 3 was model 2 additionally adjusted for waist circumference-to-height ratio. Odds ratios were computed from the generalized linear mixed-effect model with logit-link. A 2-sided *P*-value < .05 was considered statistically significant and are bolded. Multiple testing was corrected with Sidak's correction. Multiple imputations were used to account for missing variables. Categorical predictor level relative to the reference category is associated with the odds of the outcome. Transient elastography cut-off values were used for fibrosis staging [F0-F4: F0-F1, < 7.9 kPa; F2, 7.9-<8.8 kPa; F3, 8.8-<11.7 kPa; and F4 (cirrhosis), ≥ 11.7 kPa]. Cut-off values for controlled attenuation parameter score for different grades of steatosis (S0-S3) were derived from a meta-analysis on controlled attenuation parameter technology: S0 was defined as a score of less than 248 dB/m (<10% steatosis); S1 as a score of 248 to less than 268 dB/m [10- < 33% steatosis (mild)]; S2 as a score of 268 to less than 280 dB/m [33-< 66% steatosis (moderate)]; and S3 as a score of 280 dB/m or more [ ≥ 66% steatosis (severe)]. In children and adolescents ≤19 years of age, underweight (<5th percentile) and normal weight categories were combined due to few samples in the underweight category (n = 98). Body mass index-assessed normal weight, overweight, and obesity in >19-year-old participants were categorized as <25 kg/m^2^, ≥ 25 kg/m^2^, and ≥30 kg/m^2^, respectively. For participants aged <19 years, the Centers for Disease Control growth chart of >85th to <95th percentile was categorized as overweight and ≥95th percentile as obesity.

Abbreviation: CI, confidence interval.

Among adults aged >19 years, relative to the normal weight category, the overweight category predicted higher odds of liver steatosis (8.23 [6.18-10.96] *P* < .001) and fibrosis/cirrhosis (1.63 [1.07-2.47] *P* = .022) in a fully adjusted model (Table 6). Further adjustment for waist-to-height ratio significantly attenuated the strength of the association between overweight and liver steatosis 2-fold (4.13 [2.99-5.72] *P* < .001), while the association between overweight and liver fibrosis was reversed (.31 [0.19-0.71] *P* < .001). Relative to the normal weight category, the obesity category predicted higher odds of liver steatosis (21.98 [16.95-28.51] *P* < .001) and fibrosis/cirrhosis (15.01 [10.09-22.31] *P* < .001) in a fully adjusted model in >19-year-olds (Table 6). Further adjustment for waist-to-height ratio significantly attenuated the strength of the association between obesity and liver steatosis by 4.6-fold (4.77 [3.14-7.26] *P* < .001), while the association between obesity and liver fibrosis was reversed (.37 [0.19-0.71] *P* = .003).

**Figure 3.**
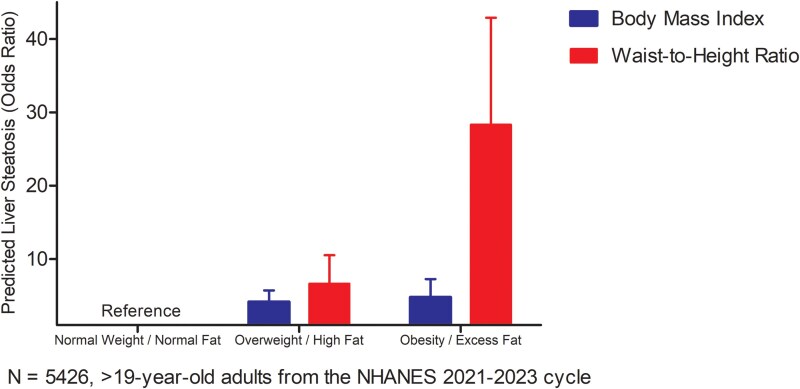
Comparison waist circumference-to-height ratio and body mass index in predicting liver steatosis. Data was drawn from the US National Health and Nutrition Examination Survey (conducted between August 2021 and August 2023). Altogether, 5426 participants were included in the analysis. Model was adjusted for age, sex, systolic blood pressure, heart rate, educational status, smoking status, race, sedentary time, moderate physical activity, and fasting insulin, glucose, total cholesterol, and high sensitivity-C-reactive protein and additionally adjusted for either waist circumference-to-height ratio or body mass index depending on the predictor. Odds ratios were computed from the generalized linear mixed-effect model with logit-link. A 2-sided *P*-value < .05 was considered statistically significant. Multiple testing was corrected with Sidak's correction. Multiple imputations were used to account for missing variables. Categorical predictor level relative to the reference category is associated with the odds of the outcome. Cut-off values for CAP score for different grades of steatosis (S0-S3) were derived from a meta-analysis on CAP technology: S0 was defined as a score of less than 248 dB/m (<10% steatosis); S1 as a score of 248 to less than 268 dB/m [10–< 33% steatosis (mild)]; S2 as a score of 268 to less than 280 dB/m [33–< 66% steatosis (moderate)]; and S3 as a score of 280 dB/m or more [ ≥ 66% steatosis (severe)]. BMI-assessed normal weight, overweight, and obesity in >19-year-old participants were categorized as <25 kg/m^2^, ≥ 25 kg/m^2^, and ≥30 kg/m^2^, respectively. Waist-to-height ratio cut points were <0.50 (normal fat mass), 0.50 to < 0.53 (high fat mass), and >0.53 (excess fat mass) [15, 16]. The raw data for odd ratios for BMI overweight and obesity categories in relation to liver steatosis are presented in Table 6. Among adults aged >19 years, relative to the normal fat waist-to-height ratio category, the high fat and excess fat waist-to-height ratio categories predicted higher odds of liver steatosis (6.64 [4.18-10.53] *P* < .001) and (28.31 [18.68-42.90] *P* < .001), respectively. Abbreviations: BMI, body mass index; CAP, controlled attenuation parameter; CI, confidence interval.

Waist-to-height ratio categories of high fat mass and excess fat mass separately predicted a higher odds of liver steatosis significantly better (1.6-fold and 6-fold, respectively) than BMI overweight and obesity after full covariate adjustments and mutual adjustment for BMI or waist-to-height ratio depending on the predictor (Fig. 3).

## Discussion

In a large multiracial US population with diverse age groups, the newly developed waist-to-height ratio cut points [16] predicted the risk of liver steatosis and fibrosis in a dose-response manner. Participants with an excess fat mass waist-to-height ratio had nearly 3-fold higher odds of liver steatosis compared to those in high fat mass waist-to-height ratio categories. The findings were consistent across the races and age groups studied, suggesting that waist-to-height ratio cut points can be useful clinically and for public health screening, prevention, diagnosis, and management of obesity and its sequelae [1, 2, 16]. The associations between WHtR and liver fibrosis were significant among older but not younger participants. The positive associations of BMI obesity categories with liver steatosis and fibrosis were significantly diminished or completely reversed when waist-to-height ratio was controlled for.

Metabolic-associated steatotic liver disease (MASLD) could progress into steatohepatitis, fibrosis, cirrhosis, and eventually hepatocellular carcinoma [22]. MASLD has been associated with chronic kidney diseases, cardiovascular disease, and mortality [22-24]. The prevalence of liver steatosis is 20% while liver fibrosis is 2.5% in young adulthood, but in the present population the prevalence of liver steatosis is 12% and liver fibrosis is 27% [20]. This suggests a significant adverse progression from liver steatosis to fibrosis in the US population, likely due to a significantly higher prevalence of obesity compared to a younger and healthier cohort [16]. Among 121 Finnish adults with type 1 diabetes, with a median age of 38.5 years and a 21-year diabetes duration, a waist-to-height ratio cutoff of 0.5 had a higher sensitivity (86%) than BMI of 26.6 kg/m^2^ with a sensitivity of 79% in detecting the risk of liver steatosis [10]. A systematic review and meta-analysis of 27 studies, which included 93 536 participants, concluded that waist-to-height ratio had an 82% predictive accuracy for detecting MASLD [17].

A recent study among 4444 participants diagnosed with MASLD from the NHANES third cycle survey conducted from 1988 to 1994 reported that waist-to-height ratio as a continuous variable was a stronger predictor of mortality than BMI [11]. The authors further examined waist-to-height ratio quartile cut points of 0.35 to < 0.53 (quartile 1), 0.53 to 0.60 (quartile 2), 0.60 to 0.66 (quartile 3), and >0.66 (quartile 4) with the risk of mortality [11]. Relative to the first quartile, the fully adjusted mortality risk (hazard ratio) for the second quartile was 1.06 (1.06-1.06), the third quartile was 1.38 (1.37-1.38), and the fourth quartile was 1.88 (1.88-1.87) [11]. These quartile waist-to-height ratio values are significantly higher than the waist-to-height ratio cut points derived in a healthy cohort, indicating that severe obesity class I to III was present in participants with MASLD [11, 16]. For example, the waist-to-height ratio 25th percentile of participants with MASLD was 0.53, which is similar to the 95th percentile (0.53) in healthy male youth [11, 15, 16]. The 0.53 cutoff in healthy male youth had a 0.94 area under the curve for detecting DEXA-measured total body fat mass and trunk fat mass [16].

In 1996, waist-to-height ratio was identified as the best surrogate anthropometric measure for cross-sectionally predicting computed tomography intraabdominal fat mass in 47 participants [25]. In 2024, waist-to-height ratio was identified as the best surrogate for longitudinally predicting both DEXA-measured total body fat mass and trunk fat mass in >7000 participants followed up for 15 years with a predictive accuracy of 89% [16]. In the secondary analysis of 2 multiracial double-blind randomized controlled trials, waist-to-height ratio was superior to BMI, waist circumference, and waist-to-hip ratio in predicting the risk of heart failure and hospitalization and showed no evidence for an “obesity-survival paradox” previously reported with BMI [14, 26]. In the present study, the association of BMI-overweight and obesity with liver steatosis attenuated 1.6-fold and 3.3-fold, respectively, after controlling for waist-to-height ratio. Importantly, the positive association between BMI-obesity and liver fibrosis was reversed, suggestive of a protective effect. The liver steatosis predictive ability of waist-to-height ratio excess fat was 6-fold larger than the predictive ability of BMI-obesity. These findings support recent evidence that waist-to-height ratio is a better detector of adverse effects of adiposity on the liver, pancreas, and heart compared to BMI [14-16]. Given the several limitations of BMI, the Lancet Commission on redefining and diagnosing obesity and the European Association for the Study of Obesity's framework for diagnosis, staging, and management of obesity have strongly recommended that the clinical assessment of obesity requires confirmation of excess/abnormal adiposity by a direct body fat measurement, such as DEXA, or by at least 1 anthropometric criteria such as waist-to-height ratio in addition to BMI [1, 2, 9]. The currently validated waist-to-height ratio pediatric cut point further enhanced the recommendations and clinical usefulness [1, 2, 15, 16].

Weight-based bias and stigma present a major obstacle in efforts to effectively prevent and manage obesity, especially in the young population [1]. Tackling stigma is both a social justice and a means to prevent obesity sequelae and mortality [1, 3]. However, 1 of the reinforcers of overweight and obesity stigma is derived from the weight-based name of the disease, which is largely due to BMI assessment. A recent prospective study reported that two-thirds of children identified as BMI-overweight have a normal waist-to-height ratio fat mass [15]. This misidentification of children may result in unnecessary intervention and stigmatization [15]. Since several consensus statements and clinical guidelines are strongly emphasizing a shift away from BMI, it is also important to reconsider the nomenclature of the disease [1, 2, 4]. Obesity, defined as excess adiposity, could be appropriately referred to as adiposopathy (sick fat), which was coined over 2 decades ago [27]. Adiposopathy is defined as “pathogenic adipose tissue anatomic/functional derangements, promoted by positive caloric balance in genetically and environmentally susceptible individuals, that result in adverse endocrine and immune responses that directly and/or indirectly contribute to metabolic diseases (eg, type 2 diabetes mellitus, hypertension, dyslipidemia, cardiovascular disease, and cancer). Adiposopathy is analogous to the disease state of other body organs, such as cardiomyopathy, encephalopathy, retinopathy, nephropathy, and neuropathy” [9].

BMI is not fat mass-specific and is an incorrect tool for assessing adiposopathy [5, 16, 28]. Since waist-to-height ratio is fat mass-specific, it is rational to consider waist-to-height ratio as an inexpensive and universally accessible surrogate marker of excess adiposity (adiposopathy) [15, 16]. With the newly developed waist-to-height ratio cut points [15, 16], a high fat mass waist-to-height ratio among males (0.50-< 0.53) could be considered adiposopathy grade I, while an excess waist-to-height ratio (≥0.53) could be considered adiposopathy grade II [15]. This new pediatric-based waist-to-height ratio estimated fat mass cutoff was recently shown to predict the risk of prediabetes and type 2 diabetes in adults [15]. Adopting this nomenclature (adiposopathy) in clinical guidelines may contribute to decreasing the stigma associated with obesity and may also help improve the International Classification of Diseases, based on 3 dimensions: etiology, degree of obesity, and health risks [1, 4, 9].

### Strength and Limitations

In this large multiracial population spanning childhood/adolescence to old age, the newly developed waist-to-height ratio pediatric cutoffs from a healthy and largely lean British cohort were externally validated. Some limitations include the unavailability of a direct fat mass assessment such as a DEXA scan), liver biopsy, and liver enzyme assays since they were not measured during the 2021 to 2023 NHANES survey. The use of FibroScan ultrasound scan to assess liver fibrosis in participants with abdominal obesity may limit the sensitivity of the assessment, necessitating a more accurate assessment of the liver. The lack of data from the Asian population warrants further external validation studies in this racial group. A recent adult study suggests that Asians may have different waist-to-height ratio cutoffs, compared to Whites; for instance, 86% of Whites had a WHtR of >0.6 compared to only 7.8% of Asians [14]. Future studies could validate the new waist-to-height ratio cut points against the risk of cardiovascular and all-cause mortality since these variables were not available in the current analysis. Missing covariates could bias the results and reduce statistical power; hence we conducted multiple imputations to account for data missingness [29]. Lastly, establishing causation with observational studies is challenging and warrants further experimental studies.

## Conclusion

The new waist-to-height ratio cut points [15, 16] strongly predicted the risk of liver steatosis and fibrosis in a dose-response manner similarly across races, sex, and age groups. The waist-to-height ratio pediatric cut points used in this study were developed in 9- to 24-year-olds but are applied to the same population, of which four-fifths are over the age of 24 years. Importantly, the cut points were validated for a hepatic disease that is less common in the pediatric population. The odds of waist-to-height ratio excess fat in predicting liver steatosis is 6-fold higher than BMI-obesity. The simple and universally accessible waist-to-height ratio cut points may be useful in clinical and public health practice for obesity screening, prevention, diagnosis, and management. A freely accessible waist-to-height ratio calculator can be used in primary care centers, homes, offices, and clinics to inexpensively confirm BMI-based obesity diagnosis where an expensive direct assessment of excess fat such as DEXA is lacking [1, 2].

## Data Availability

Original data generated and analyzed during this study are included in the data repositories listed in the References.
